# Polypharmacy, salivary function, and oral health outcomes in institutionalized older adults: A scoping review

**DOI:** 10.4317/jced.63940

**Published:** 2026-03-30

**Authors:** Fernando Mauricio Espada-Salgado, María Mihaela Iuga, Bogdan Oprea, Alex Rodrigo A. Capellino-Gambetta

**Affiliations:** 1 Faculty of Health Sciences, Private University of Tacna, Tacna, Peru; 2 Department of Dental Morphology, University Lucian Blaga Sibiu, Sibiu, Romania

## Abstract

**Background:**

Polypharmacy is common in long-term care facilities (LTCFs) and may contribute to xerostomia, hyposalivation, and oral disease. However, evidence specific to LTCF populations has not been comprehensively mapped. Objective: To map the available evidence on the association between polypharmacy or medication burden and salivary dysfunction and oral health outcomes in institutionalized older adults.

**Material and Methods:**

This scoping review was conducted in accordance with Joanna Briggs Institute guidance and reported following PRISMA-ScR. PubMed/MEDLINE, Embase, Scopus, Web of Science Core Collection, and LILACS via BVS were searched from 1 January 2005 to 23 January 2026. To identify potentially relevant grey literature, Google Scholar and ClinicalTrials.gov were also searched. Screening was performed in Rayyan, data were extracted using a piloted charting form, and findings were synthesized narratively.

**Results:**

Eight studies published between 2010 and 2025 were included. Seven used a cross-sectional design and one was a randomized controlled trial, all conducted in LTCF settings in Europe and Asia. Polypharmacy, commonly defined as the use of five or more medications, was highly prevalent. Measures of xerogenic or anticholinergic burden identified associations with xerostomia or reduced salivary flow more consistently than medication count alone. Dry mouth was frequent, and where oral outcomes were assessed, substantial oral morbidity was also reported.

**Conclusions:**

In institutional care settings, salivary dysfunction and oral disease are common and appear to be more closely associated with xerogenic or anticholinergic burden than with medication number alone. Preventive oral care protocols integrated with xerogenic risk-informed medication review and standardized outcome assessment should be prioritized in future LTCF research and care.

## Introduction

Population ageing and the rising prevalence of multimorbidity have increased the complexity of pharmacotherapy in older adults, especially among those living in long - term care facilities. In this context, polypharmacy is common and clinically relevant, although definitions vary. The most frequently used operational definition is a numerical threshold, often five or more concurrent medications (1 , 2). At the population level, polypharmacy affects a substantial proportion of older adults and reflects both clinical complexity and prescribing patterns (3). In residential aged care, residents often use even more medications than older people living at home, making medication burden a central and potentially modifiable determinant of adverse outcomes, including oral complications (4). Saliva is essential for oral homeostasis through lubrication, buffering, antimicrobial activity, and support of remineralization. Medication-related dry mouth is a frequent problem in older adults, encompassing xerostomia as a subjective symptom and hyposalivation as objectively reduced salivary flow. A systematic review and meta-analysis in older populations found that medication use is significantly associated with xerostomia and salivary gland hypofunction, with increased risk observed across several therapeutic classes (5). Beyond discomfort, salivary hypofunction is clinically relevant because it can contribute to dental caries including root caries, periodontal deterioration, mucosal discomfort, denture intolerance, opportunistic infections, impaired nutrition, and reduced quality of life (6). Medication burden is not limited to the number of drugs. Burden indices, particularly anticholinergic burden, provide a mechanistic link between prescribing patterns and salivary dysfunction because anticholinergic effects reduce salivary secretion. Empirical evidence shows that high anticholinergic burden is associated with xerostomia and low unstimulated salivary secretion in older adults (7). Additional research suggests that high anticholinergic burden may be more strongly associated with objective hyposalivation, both unstimulated and stimulated, than with xerostomia symptoms alone (8). This highlights the importance of assessing both symptom-based and measurement-based salivary outcomes when evaluating medication effects. Oral health outcomes linked to xerogenic medication exposure extend beyond salivary function. Longitudinal register-based evidence in people with dementia has demonstrated a dose-response relationship between the number of xerostomic medications and tooth extraction risk, supporting the clinical significance of sustained medication induced salivary dysfunction (9). Similarly, survey data in dependent older adults show that xerostomia is associated with polypharmacy and specific medication patterns, reinforcing the relevance of medication burden in vulnerable older groups who often overlap with institutional care populations (10). Despite the growing evidence, important gaps remain in understanding how polypharmacy and overall medication burden influence salivary function and oral health outcomes in institutionalized older adults. Many studies focus on broader older populations or assess limited medication groups, and relatively few integrate xerostomia, objective hyposalivation, and oral disease outcomes specifically in long - term care residents (4 , 6). Therefore, this scoping review aims to systematically map the literature from 2005 onward in English, Spanish, and Portuguese, describing the magnitude of polypharmacy among long - term care residents and synthesizing evidence on its associations with salivary dysfunction and oral health outcomes. This work will help identify knowledge gaps, guide future research, and support interdisciplinary strategies to improve oral health and quality of life in this vulnerable population.

## Material and Methods

- Methodological approach This scoping review was conducted in accordance with the Joanna Briggs Institute (JBI) methodology for scoping reviews and is reported following the Preferred Reporting Items for Systematic Reviews and Meta-Analyses extension for Scoping Reviews (PRISMA-ScR) (11 , 12). In keeping with scoping review methodology, no formal critical appraisal or risk-of-bias assessment was performed, as the aim was to map the extent, nature, and characteristics of the available evidence rather than to estimate pooled effects or determine certainty of evidence (11). - Protocol and registration An a priori protocol was developed before study selection. The protocol specified the review question, the Population-Concept-Context (PCC) framework, eligibility criteria, information sources, search procedures, screening workflow, and data-charting variables. The protocol and all supporting materials, including the full search strategies and screening logs, are available in the Open Science Framework (OSF) repository: https://osf.io/qnf5r - Review question and PCC framework The review question was formulated using the PCC framework. Population: Institutionalized older adults, preferably aged 60 years or older, or described as geriatric by the study authors (for example, studies with a mean or median age of 65 years or older), residing in long-term care facilities such as nursing homes, residential care homes, care homes, skilled nursing facilities, geriatric institutions, ILPI, or equivalent settings. Concept: Polypharmacy and/or medication burden, including author-defined polypharmacy thresholds (for example, five or more medications), medication burden indices (for example, Drug Burden Index, Anticholinergic Cognitive Burden, and Anticholinergic Risk Scale), anticholinergic burden or load, xerogenic risk scores, and their association with salivary function outcomes and/or oral health outcomes. Salivary outcomes included xerostomia, hyposalivation, salivary hypofunction, salivary flow rate, and sialometry-based measures. Oral health outcomes included dental caries, including root caries, periodontal outcomes, oral mucosal lesions, oral infections such as candidiasis and denture stomatitis, oral hygiene indicators, tooth loss, and related clinical indices. Context: Long-term care or other institutional settings. Studies of community-dwelling older adults were considered only when data for institutionalized participants were reported separately. The review question was: What evidence published since 2005 describes polypharmacy or medication burden among institutionalized older adults and its association with salivary dysfunction and oral health outcomes in long-term care settings? - Eligibility criteria Inclusion criteria Studies were considered eligible when they met all of the following criteria: Participants: Institutionalized older adults living in long-term care facilities. Studies were eligible if the sample consisted predominantly of older adults (for example, aged 60 years or older) or was explicitly described as geriatric. Exposure or key concept: Polypharmacy or a measurable proxy of medication burden, including number of medications, medication burden indices, anticholinergic burden, or anticholinergic load. Outcomes: At least one salivary function outcome and/or one oral health outcome, with an explicit relationship to medication exposure or polypharmacy. Types of evidence: Primary quantitative studies, including cross-sectional, cohort, case-control, clinical trial, and quasi-experimental designs. Qualitative or mixed-methods studies were also eligible if they reported salivary and/or oral health outcomes in relation to medication exposure. Grey literature, such as theses and trial registry records, was considered when sufficient primary data were available. Review articles were used only for background and backward citation searching and were not included as evidence sources. Time frame and languages: Studies published from 1 January 2005 to 23 January 2026 in English, Spanish, or Portuguese. Exclusion criteria Studies were excluded when they met any of the following criteria: Wrong context: Community-dwelling older adults only, with no institutional subgroup or with non-separable institutional data; acute inpatient hospital settings unless they clearly represented long-term residential care and reported separable data. Wrong concept: Medication use was mentioned, but polypharmacy or medication burden was not defined or measured. Wrong outcomes: No salivary function outcomes and no oral health outcomes were reported. Publication type and other exclusions: Editorials, letters, commentaries, and protocols without results; conference abstracts or proceedings without a peer-reviewed full text; animal or in vitro studies; pediatric or non-older adult samples; and single case reports. - Information sources and search strategy A systematic search was conducted in five bibliographic databases: Embase, Scopus, Web of Science Core Collection, PubMed/MEDLINE, and LILACS via the Virtual Health Library (BVS). To broaden coverage and identify potentially relevant grey literature, supplementary searches were also conducted in Google Scholar and the ClinicalTrials.gov registry. The search strategy combined controlled vocabulary, when available (for example, MeSH, Emtree, and DeCS), with free-text terms tailored to each source. The strategy was structured around three core domains: (1) polypharmacy and medication burden, including anticholinergic burden/load and named indices; (2) salivary dysfunction outcomes, including xerostomia, dry mouth, hyposalivation, salivary hypofunction, sialometry, and salivary flow; and (3) institutional long-term care settings, including nursing homes, residential care homes, care homes, skilled nursing facilities, and institutionalized older adults. To maximize sensitivity, salivary outcome terms were searched in title and abstract fields and through controlled vocabulary when available, rather than being restricted to title fields only. All searches were limited to the period from 1 January 2005 to 23 January 2026. The final search update was run on 23 January 2026. Because Google Scholar retrieves a high volume of less structured results, a simplified search strategy based on the main concepts of the review was used. Google Scholar was searched on 23 January 2026 using the following query: (xerostomia OR "dry mouth" OR hyposalivation OR "salivary hypofunction") ("nursing home" OR "long-term care" OR "residential care" OR LTCF) (polypharmacy OR "anticholinergic burden" OR "medication burden") (thesis OR dissertation OR "doctoral thesis" OR "PhD thesis") filetype:pdf -radiotherapy -cancer. All 109 records retrieved by this search at the time of execution were screened for eligibility. ClinicalTrials.gov was searched on 23 January 2026 in Expert Search mode using the following query: ("anticholinergic burden" OR "drug burden" OR "medication burden" OR polypharmacy) AND (frail OR elderly OR "older adults" OR geriatric) AND ("nursing home" OR "long-term care" OR residential OR institutional) AND AREA[StudyFirstPostDate]RANGE[01/01/2005, MAX]. All 8 retrieved trial registry records were screened using the same eligibility criteria applied to the bibliographic sources. In addition, the reference lists of all included studies and relevant review articles were hand-searched to identify further eligible records through backward citation searching. Full source-specific search strategies, supplementary search procedures, and screening logs are available in the OSF repository (https://osf.io/qnf5r). An example of the PubMed/MEDLINE strategy, adapted for the other sources, was as follows: ("Polypharmacy"[Mesh] OR polypharmacy[tiab] OR "drug burden"[tiab] OR "medication burden"[tiab] OR "anticholinergic burden"[tiab] OR "anticholinergic load"[tiab] OR "Drug Burden Index"[tiab] OR "Anticholinergic Cognitive Burden"[tiab] OR "Anticholinergic Risk Scale"[tiab]) AND ("Xerostomia"[Mesh] OR xerostomia[tiab] OR "dry mouth"[tiab] OR hyposalivation[tiab] OR "salivary hypofunction"[tiab] OR "salivary gland hypofunction"[tiab] OR "salivary flow"[tiab] OR sialometr*[tiab] OR saliva*[tiab]) AND ("Nursing Homes"[Mesh] OR "Long-Term Care"[Mesh] OR institutional*[tiab] OR "nursing home*"[tiab] OR "long-term care"[tiab] OR "residential care"[tiab] OR "care home*"[tiab] OR "skilled nursing"[tiab] OR ILPI[tiab]). - Selection of sources of evidence Records retrieved from all sources were exported to reference management software for de-duplication and then imported into Rayyan for screening (13). Two reviewers independently screened titles and abstracts, followed by independent full-text assessment of potentially eligible reports. Disagreements were resolved through discussion and consensus, with consultation of a third reviewer when necessary. Reasons for exclusion at the full-text stage were recorded prospectively. The study selection process is summarized in the PRISMA-ScR flow diagram. Across bibliographic databases and supplementary sources, 210 records were identified, including 93 from bibliographic databases and 117 from other sources. After removal of duplicates (n = 26), 184 unique records remained for title and abstract screening. Of these, 11 reports were sought for retrieval, all of which were obtained and assessed in full text. Three reports were excluded after full-text review: one because institutional data were not reported separately, and two because no eligible exposure-outcome association was reported. Eight studies were therefore included in the final data charting and synthesis. - Data charting A piloted data-charting form was used to extract the following information from each included study: author and year; country and setting; type of facility; study design; participant characteristics; operational definition of polypharmacy or medication burden, including thresholds and indices; medication classes of interest, particularly xerogenic and anticholinergic agents; salivary outcomes, including xerostomia measures, hyposalivation criteria, sialometry, and salivary flow; oral health outcomes, including caries and root caries, periodontal indices, mucosal lesions, candidiasis, denture stomatitis, oral hygiene indices, and tooth loss; analytical approach; and key findings on the association between medication burden, salivary dysfunction, and oral health outcomes. One reviewer performed the initial data charting and a second reviewer checked all entries for accuracy and completeness. Any discrepancies were resolved by consensus. - Synthesis of results The findings were synthesized descriptively and presented in narrative form and summary tables. The evidence was organized according to: (1) exposure definition, distinguishing simple polypharmacy thresholds from medication burden indices; (2) salivary outcomes, distinguishing subjective xerostomia from objective hyposalivation or measured salivary flow; and (3) oral health outcomes, including caries and root caries, periodontal outcomes, mucosal and infectious conditions, oral hygiene indicators, and tooth loss. Because of the heterogeneity in study designs, exposure definitions, salivary measures, and oral health outcomes, no meta-analysis was planned or conducted. - Data availability All supporting materials, including the full search strategies, supplementary search details, screening logs, PRISMA-ScR flow information, and data-charting templates, are available in the Open Science Framework (OSF) repository: https://osf.io/qnf5r

## Results

**Figure 1:**
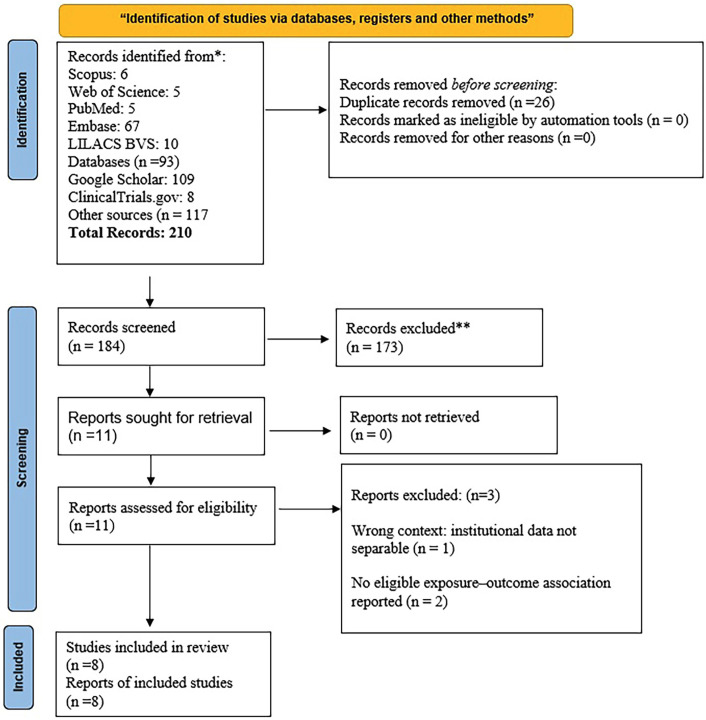
PRISMA-ScR flow diagram of study identification and selection from bibliographic databases and supplementary sources. Searches were last updated on 23 January 2026.

- Study Selection A total of 210 records were identified across bibliographic databases and supplementary sources, including 93 records from databases and 117 from other sources. After duplicate removal (n = 26), 184 unique records remained for title and abstract screening. Of these, 173 were excluded. Eleven reports were retrieved and assessed in full text. Three reports were excluded at this stage: one because institutional data were not reported separately, and two because no eligible exposure-outcome association was reported. Consequently, eight studies were included in this scoping review, (Fig. 1).

1

- Characteristics of Included Studies The eight included studies were published between 2010 and 2025 and were conducted in Europe (France, the Netherlands, Belgium, Switzerland, Poland, Lithuania, and Norway) and Asia (Singapore). All studies were performed in institutional settings, mainly nursing homes or long-term geriatric care wards. Seven studies used an observational cross-sectional design, and one was a randomized controlled trial. Sample sizes ranged from 50 to 1,226 participants. Mean ages were generally between 75 and 85 years. When reported, women represented the majority of participants, usually accounting for approximately 60% to 75% of the sample, which is consistent with the demographic profile of long-term care populations. Table 1 summarizes the main characteristics of the included studies.

Table 1

Several studies went beyond simple medication counts and assessed medication burden using xerogenic or anticholinergic frameworks. Two main approaches were identified. First, anticholinergic burden was quantified using the Anticholinergic Drug Scale (ADS); for example, Kersten et al. included residents with an ADS score of 3 or higher. Second, xerostomic risk was estimated using medication-based scoring systems, as in Janssens et al., where each drug was weighted according to its xerogenic potential and then summed into an individual risk score. In that study, approximately half of all recorded medications were classified as potentially hyposalivary, with participants using an average of 4 to 5 xerogenic medications each. Across the included studies, the drug classes most frequently implicated were anticholinergics, cardiovascular agents (particularly antihypertensives and diuretics), and psychotropic medications, including antidepressants, antipsychotics, anxiolytics, and sedatives. However, at least one study suggested that dry-mouth risk was more strongly associated with the overall number of medications than with any single drug class. Table 2 summarizes how each study operationalized polypharmacy or medication burden and which indices or medication categories were emphasized.

Table 2

- Salivary Function Outcomes (Xerostomia and Hyposalivation) Across the included studies, the reported prevalence of dry mouth varied according to the assessment method used. When symptom-based measures were applied, xerostomia was reported in 37.3% of residents in French geriatric wards and in about 52% of Dutch nursing home residents based on the Xerostomia Inventory. By contrast, the Singapore nursing home study reported dry mouth in 63.6% of participants using the Clinical Oral Dryness Score (CODS), suggesting that clinical sign-based measures may yield higher prevalence estimates than symptom-based instruments. In Poland, a study that combined questionnaires such as Fox's test with a mirror sliding test also documented frequent complaints of dry mouth and found that higher medication counts were associated with greater dryness and more reports of "lack of saliva" symptoms (p = 0.036 and p = 0.009, respectively). Objective assessment of salivary flow or hyposalivation was less frequently reported but provided additional clinical insight. In the Dutch sample, 24% of participants met the criterion for unstimulated hyposalivation, and up to 60% had reduced stimulated salivary flow when standard cutoffs were applied, such as less than 0.1 mL/min for unstimulated flow. Mean unstimulated salivary flow was also lower than that observed in a younger reference group. In the only deprescribing trial, Kersten et al. measured salivary output using an absorbent swab method and found no significant improvement in either salivary flow or subjective oral dryness after reduction of anticholinergic burden at 4 or 8 weeks. These findings suggest that salivary hypofunction may show limited short-term reversibility in frail long-term care residents. Regarding the relationship between medication exposure and salivary outcomes, most studies supported an association between greater medication burden and poorer salivary status, although the relative importance of medication count and medication profile varied across studies. Some findings suggested an additive effect of medication number, with residents taking more drugs showing greater clinical oral dryness. In contrast, the French cohort emphasized medication profile rather than medication count, showing that anticholinergic exposure increased the odds of xerostomia (OR 1.35, 95% CI 1.05 to 1.73), whereas polypharmacy defined as five or more medications was not independently associated with the outcome. Similarly, in the Singapore study, where polypharmacy was nearly universal, a simple medication threshold did not discriminate dry mouth risk. This may reflect the limited variability in medication counts as well as the use of an objective clinical dryness score. Overall, the evidence suggests that salivary dysfunction is common in institutionalized older adults and is generally associated with medication burden. Xerogenic or anticholinergic load may be a more sensitive predictor than medication count alone, particularly in highly medicated long-term care populations. - Oral health outcomes (caries, periodontal status, tooth loss, and oral symptoms) Although oral assessments varied across the included studies, the available evidence consistently points to a high burden of oral disease among institutionalized older adults and suggests clinically relevant associations with medication exposure and dry mouth. Dental caries and tooth loss were frequently reported. In Singapore nursing homes, just over half of residents had at least one untreated carious lesion, whether coronal or root related. European cohorts also showed substantial cumulative disease, reflected in extensive tooth loss and high levels of edentulism. In Janssens et al., among dentate residents, a higher xerogenic medication burden was associated with fewer remaining natural teeth (p = 0.005) and a higher restorative or treatment index. This pattern may partly explain the lower number of observable untreated carious teeth in heavily medicated individuals, as the most susceptible teeth may already have been lost. This interpretation is consistent with the high prevalence of edentulism reported in that cohort, which was approximately 59%. Similarly, a Lithuanian pilot study found an inverse association between medication count and tooth retention (r -0.406). In the Swiss nursing home study, tooth loss was also substantial, with a mean of about 14 missing teeth, although differences according to polypharmacy status were difficult to detect because polypharmacy was nearly universal. Root caries appeared to be particularly relevant. In Singapore, dry mouth was independently associated with root surface caries (OR 1.93, p &lt; 0.05), which is consistent with the protective role of saliva on exposed root surfaces. Findings related to root conditions were less consistent in the other studies. However, the Swiss cohort reported frequent residual root fragments and a tendency toward higher counts among residents taking xerogenic drug classes, although these associations were not uniformly statistically significant. Periodontal proxies and oral hygiene indicators suggested inadequate daily oral care in long-term care facilities. Detailed periodontal examination was generally lacking, which represents an important evidence gap. Nevertheless, the Singapore study reported very high levels of gingival inflammation, affecting approximately 89% of participants, together with substantial plaque accumulation. Dry mouth may further worsen oral hygiene difficulties, as residents with clinical oral dryness were more likely to present heavy plaque deposits (OR 5.09, p &lt; 0.01). This supports a plausible pathway in which medication-related salivary dysfunction contributes to plaque retention and inflammation, thereby increasing susceptibility to caries and periodontal deterioration. Mucosal and functional outcomes were reported less consistently, but they remain clinically relevant. In a Polish study of institutional care residents, greater oral dryness was associated with burning mouth (p = 0.026) and swallowing difficulty (p = 0.037), whereas taste disturbance was associated with polypharmacy (p = 0.041). Denture related difficulties also increased with longer institutional stay, which is consistent with the clinical impact of salivary dysfunction on comfort and prosthesis tolerance, even when specific mucosal diagnoses are not systematically recorded. Overall, the evidence suggests substantial cumulative oral morbidity among institutionalized older adults, especially tooth loss, caries, including root caries, and hygiene related inflammation. Medication exposure and dry mouth appear to contribute through both salivary dysfunction and reduced oral function and hygiene. Table 3 summarizes the oral health outcomes assessed and the direction and magnitude of the reported associations with polypharmacy, xerogenic exposure, or clinical oral dryness.

Table 3

## Discussion

Polypharmacy is a common and growing challenge in the care of institutionalized older adults. Although definitions vary, it is most often described as the regular use of five or more medications, a threshold that does not account for the xerogenic potential of specific drugs or drug combinations (1 - 3). In long-term care facilities (LTCFs), the high burden of multimorbidity and dependence makes complex prescribing difficult to avoid. As a result, many residents are exposed to multiple medications with xerogenic or anticholinergic properties, creating a plausible iatrogenic pathway to salivary dysfunction and subsequent oral complications (4 - 6). This scoping review therefore examined how medication burden relates to salivary function and oral health in LTCFs. - Evidence from long-term care studies Eight LTCF studies were mapped. A cross-sectional study conducted in Singapore nursing homes suggested that oral dryness may act as an intermediary factor linking systemic disease burden with poorer oral outcomes, and also indicated that objective clinical signs of dry mouth may be more sensitive than residents' self-reports (14). A French multicenter study in geriatric wards found that xerostomia was more strongly associated with anticholinergic medication exposure than with medication count alone, highlighting the importance of medication profile when assessing risk (15). A Dutch study in nursing home residents showed that subjective xerostomia and objective hyposalivation overlap but are not interchangeable, supporting the value of combining symptom-based screening with salivary flow assessment in frail older populations (16). Dental status was explored in a Swiss nursing home cohort in which polypharmacy and multimorbidity were almost universal. Residents with more complex medication regimens tended to have greater tooth loss and more residual root fragments, suggesting a pattern of cumulative oral morbidity along frailty-related care trajectories (17). In the only interventional study, pharmacist-led deprescribing reduced anticholinergic burden but did not produce significant short-term improvements in salivary flow or mouth dryness, indicating that reversal of chronic xerogenic effects may be difficult in frail older adults (18). A Polish study reported frequent mucosal and functional complaints, including burning mouth and taste disturbances, and linked these symptoms to greater medication burden (19). A Lithuanian pilot study associated salivary parameters and plaque accumulation with residents' general health and medication use, offering further support for a relationship between systemic frailty, medication burden, and oral biofilm challenges (20). Finally, a large Flemish nursing home cohort introduced a medication-related dry mouth risk score and found that this score, rather than the simple number of medications, was associated with oral health status (21). - Mechanisms and oral outcomes Taken together, these findings support the view that medication profile may be as important as medication count. Previous reviews have shown that drugs with anticholinergic or sympathomimetic effects are major contributors to dry mouth in older adults, and that cumulative anticholinergic burden is associated with xerostomia and hyposalivation (5 - 8). In LTCFs, this has important clinical implications because many residents are exposed to several xerogenic medications at the same time. Reduced salivary flow compromises buffering, remineralization, and antimicrobial protection, thereby increasing susceptibility to root caries, gingival inflammation, mucosal irritation, and denture intolerance (6). Observational evidence in people with dementia has also linked xerostomic medication exposure with accelerated tooth loss and greater oral treatment needs, which is consistent with a cumulative disease pathway under chronic salivary compromise (9). National survey data in dependent older adults further document the frequent coexistence of xerostomia and polypharmacy, reinforcing the relevance of medication burden in vulnerable groups that overlap with institutional care populations (10). - Public health relevance and care home implementation From a public health perspective, polypharmacy in LTCFs is widespread and is expected to increase as residents live longer with multiple chronic conditions (22). Ageing itself may be associated with modest reductions in resting salivary flow, which can further amplify medication-related effects in frail older adults (23). Medication-induced salivary gland dysfunction is increasingly recognized as an iatrogenic condition, and previous reviews have summarized its prevalence, diagnosis, and management (24). Meta-analytic evidence also indicates that hyposalivation affects a substantial proportion of older people, supporting the need for proactive screening and prevention (25). At the same time, care home residents across Europe often have substantial unmet oral treatment needs and limited access to routine dental care (26), while gerodontology research continues to face methodological and implementation challenges, especially in institutional settings (27). Implementation studies suggest that integrating oral care into daily care home routines is feasible when interventions are aligned with staff workflows and supported by practical training (28). For clinical assessment, structured oral dryness scoring systems linked to salivary flow and mucosal wetness may improve risk stratification and monitoring in LTCFs (29). In addition, observational work in institutionalized older adults has reported frequent sensory complaints and mucosal lesions, reminding clinicians that dryness is not only a clinical sign but also a relevant quality-of-life concern. A practical care framework for these settings should therefore include medication review, daily oral hygiene support, hydration advice, saliva substitutes or stimulants when appropriate, high-fluoride preventive measures, and early management of opportunistic infections (30 - 33). - Limitations and future directions The evidence mapped in this review has several limitations. Most included studies were cross-sectional, which limits causal inference. Definitions of polypharmacy and medication burden varied across studies, as did the methods used to assess xerostomia and hyposalivation. This heterogeneity reduces comparability across studies and limits the potential for quantitative synthesis. Oral health outcomes were also inconsistently reported, and detailed periodontal assessments were largely absent. We expanded study identification to include grey literature and trial registry sources. These supplementary searches did not yield additional eligible primary studies beyond those already included, suggesting that the LTCF evidence base remains limited and fragmented. Future research should prioritize longitudinal studies in LTCFs, standardized assessment of xerostomia and salivary flow, and pragmatic trials that combine medication review with feasible, staff-delivered oral care interventions. Greater use of implementation science will also be important to address organizational barriers and support more consistent reporting that can inform future evidence synthesis and policy translation.

## Conclusions

In conclusion, polypharmacy is highly prevalent among residents of long-term care facilities, and medications with xerogenic or anticholinergic properties appear to be important contributors to salivary dysfunction. The evidence mapped in this review suggests that dry mouth is common in this population and has meaningful clinical consequences, contributing to a range of oral problems including root caries, gingival inflammation, mucosal discomfort, and denture intolerance. Importantly, medication profile may be at least as informative as medication count, since measures of anticholinergic or xerogenic burden seem to predict xerostomia more effectively than the absolute number of medications alone. Clinicians caring for institutionalized older adults should therefore incorporate medication review informed by xerogenic risk, routine screening for dry mouth using symptom-based questions and objective measures whenever feasible, and more intensive preventive oral care for residents at higher risk. Interdisciplinary collaboration among physicians, pharmacists, dental professionals, and care home staff is essential to integrate these strategies into everyday practice. Future research should prioritize longitudinal and implementation-oriented designs to identify effective ways to reduce xerogenic burden and lessen its oral consequences. Addressing medication-related dry mouth in long-term care facilities is not only a clinical priority, but also an important public health objective in the context of healthy ageing.

## Figures and Tables

**Table 1:** Table Characteristics of the included studies (N = 8).

Study (Author, Year)	Country	Setting (Institution Type)	Design	Sample Size	Participant Demographics
Tada et al., 2025	Singapore	Nursing homes (8 facilities)	Cross-sectional	N = 173	Mean age 75.0 ± 9.1 years; 38% female
Desoutter et al., 2012	France	Long-term geriatric wards (4 wards, multicenter)	Retrospective comparative cross-sectional	N = 769	Mean age 84.6 ± 8.4 years; sex not reported
Van der Putten et al., 2011	Netherlands	Nursing home (physically impaired residents)	Cross-sectional	N = 50	Mean age 78.1 years (range 53–98); 60% female
Anliker et al., 2023	Switzerland	Geriatric nursing homes (3 facilities with integrated dental care)	Cross-sectional	N = 180	Mean age 85.5 ± 7.4 years (range 67–100); 75% female
Kersten et al., 2013	Norway	Nursing homes (22 facilities)	Randomized controlled trial (single-blind, 8 weeks)	N = 87	Mean age 85 years; 79% female
Michalak et al., 2022	Poland	Municipal Health Center for Older and Dependent People (24/7 institutional care) and Daily Medical Care House (3-month rehabilitation program)	Comparative cross-sectional	N = 80	Age reported by subgroup; 65% female (52/80)
Brukiene et al., 2011	Lithuania	Long-term care facility (Centre of Gerontology and Rehabilitation)	Cross-sectional	N = 50 institutionalized older adults	Mean age 82.0 ± 9.3 years; sex not clearly reported for the full elderly sample
Janssens et al., 2017	Belgium	Nursing homes (23 facilities; Gerodent network)	Cross-sectional	N = 1226	Mean age 83.9 ± 8.5 years; 70.0% female

In most included studies, polypharmacy was operationalized as the concurrent use of five or more medications, and this threshold was exceeded by most institutionalized residents. For example, in the Swiss geriatric nursing-home sample, 92% of participants were taking at least five medications, with a mean of 12 medications per participant. In studies reporting medication counts descriptively, mean medication use ranged from approximately 5 per participant in Lithuania to 9 in Belgium and 12 in Switzerland.

**Table 2:** Table Definitions of polypharmacy or medication burden in the included studies.

Study	Polypharmacy Definition / Measure	Medication Classes / Indices Considered
Tada 2025	Polypharmacy defined as ≥5 medications; 92.4% of participants used ≥5 medications. Analyzed as a binary variable (Yes/No, ≥5).	No specific drug classes were the main focus; the study recorded total medication count and use of xerostomia-associated medications. Polypharmacy itself was not significantly associated with dry mouth.
Desoutter 2012	Medication use assessed as the total number of current medications (mean 6; range 0–18); no explicit polypharmacy threshold was applied in the analysis.	Anticholinergic medications (identified using the Theriaque database) and xerogenic medications were specifically considered. Anticholinergic drug use was analyzed as a binary risk factor for xerostomia (yes/no), and medications associated with hypersalivation were also recorded (yes/no).
Van der Putten 2011	Medication use assessed as the total number of daily medications per resident (mean 5.3 ± 2.6; range 0–10); no explicit polypharmacy threshold was applied.	Hyposalivation-related medications were identified based on Scully’s list. The number of such medications per participant was recorded; 91 of 207 medications were classified as salivary-flow-reducing. The analysis contrasted total medication count with the subset of medications associated with reduced salivary flow rather than focusing on specific drug classes.
Anliker 2023	Polypharmacy defined as ≥5 medications. All residents met the inclusion criterion of polypharmacy and/or multimorbidity. Mean medication use was 12.1 ± 5.6 per participant (range up to 25).	Medications were grouped by class, with specific attention to drugs associated with salivary dysfunction, including antihypertensives, psychiatric medications, opioids, antihistamines, sedatives, benign prostatic hyperplasia drugs, central nervous system stimulants, and anticonvulsants. Associations with root caries and residual roots were examined
Kersten 2013	No numeric polypharmacy threshold was used. Instead, medication burden was operationalized as anticholinergic burden. Participants were eligible if they had an Anticholinergic Drug Scale (ADS) score of ≥3, indicating moderate to high anticholinergic load. Median medication use at baseline was 9 medications per participant.	The primary exposure measure was the ADS score, calculated by summing drug-specific scores from 0 to 3. Serum anticholinergic activity (SAA) was also measured as an objective indicator of total anticholinergic load. The analysis focused on overall anticholinergic burden rather than on individual drug classes.
Michalak 2022	Polypharmacy was defined as the daily use of ≥5 different medications, in accordance with the WHO definition. All participants exceeded this threshold, and medication count was also analyzed as a continuous variable.	The analysis examined the relationship between total medication count and oral complaints. No specific drug class showed a dominant effect; instead, dry mouth and taste disturbances were more closely associated with the overall number of medications than with any single medication group.
Brukiene 2011	Polypharmacy was not defined using an explicit cutoff. Medication burden was described by the mean number of medications used (5.3), alongside the mean number of systemic diseases (4.7).	No individual medication classes were analyzed because of the small sample size. The study focused on overall medication burden and examined its association with salivary function and dentition outcomes.
Janssens 2017	Polypharmacy was inferred from overall medication use rather than dichotomized using a fixed cutoff. Mean medication use was 9.0 ± 3.6, with a median of 9 medications per resident; more than 90% of participants used at least five medications. The study also emphasized an overall dry-mouth risk score based on xerogenic medication burden.	Medications with xerostomic potential were specifically identified. Overall, 49.6% of all recorded medications were classified as potentially hyposalivary. An individual “dry-mouth risk” score was calculated for each resident by summing xerogenic drug scores. ATC medication classes, including antithrombotic agents, cardiovascular drugs, diuretics, opioids, and psychoactive medications, were also examined in relation to oral health. The analysis emphasized overall xerogenic versus non-xerogenic medication burden rather than individual drug names.

2

**Table 3:** Table Summary of oral health findings and associations with medication exposure in included studies.

Study	Key Oral Health Findings (caries, periodontal, etc.)	Association with Medications (direction, magnitude)
Tada et al., 2025	50.8% had at least one untreated carious lesion (coronal and/or root); 46.6% had poor oral hygiene with visible plaque; 89.2% had gingival inflammation; 63.6% had clinical signs of oral dryness (CODS ≥1).	Polypharmacy (≥5 medications) was present in 92.4% of residents and was not significantly associated with dry mouth. However, clinical oral dryness was associated with heavier plaque accumulation (OR 5.09) and root caries (OR 1.93).
Desoutter et al., 2012	Xerostomia prevalence was 37.3% based on subjective assessment. No specific dental indices were reported, as the study focused primarily on oral dryness.	Total medication count was not a significant predictor of xerostomia. By contrast, anticholinergic medication uses increased xerostomia risk (OR 1.35, p = 0.02), whereas medications associated with hypersalivation showed a protective effect (OR 0.81, p = 0.03).
Van der Putten 2011	52% reported xerostomia; objective hyposalivation was present in 24% under unstimulated conditions and in 60% under stimulated conditions. Mean salivary flow was about 0.3 mL/min, lower than in younger controls. Plaque levels were higher in older residents; mean number of natural teeth was 5.6, and 38% were edentulous.	Higher medication count was associated with fewer remaining teeth (Pearson r = -0.406, p ≈ 0.005). The number of hyposalivation-related medications was not significantly associated with salivary flow, whereas multimorbidity was associated with lower salivary flow (r ≈ -0.40).
Anliker et al., 2023	Mean number of teeth was 14.1 ± 9.9; 14% of participants were completely edentulous. Root-related conditions were common, with 52% presenting removable dentures and many residents having at least one unrestored root remnant (mean ≈ 1.0). Age was associated with greater tooth loss, with older residents having fewer remaining teeth (p = 0.001).	Polypharmacy, defined as the use of ≥5 medications, was present in 92% of the sample and was not associated with significant differences in number of teeth or root remnants compared with residents without polypharmacy. However, residents taking medications related to dry mouth, such as antihypertensives and central nervous system stimulants, tended to have more residual roots, suggesting more untreated decay, although these associations did not reach statistical significance (p ≈ 0.06–0.09). No significant correlation was found between total medication count and current number of teeth (p = 0.253).
Kersten et al., 2013	No detailed dental examination was performed, as the study focused primarily on cognitive and salivary outcomes. At baseline, 83% of participants wore dentures, indicating a high burden of tooth loss or edentulism. Reduced salivary flow and xerostomia related side effects were common at study entry.	Reduction of anticholinergic burden, defined as a decrease of at least 2 points in ADS score, did not improve salivary flow or subjective oral dryness at 4 or 8 weeks. No significant change in oral moisture was observed after deprescribing, suggesting limited short-term reversibility of salivary dysfunction in this population. Caries and periodontal outcomes were not assessed because of the short study duration.
Michalak et al., 2022	Common oral complaints included dry mouth, mucosal burning, dysgeusia, and denture related discomfort. Mucosal burning was reported in about 25% of participants and increased with longer institutional stay (p = 0.032). Difficulty using dentures also increased with longer stay (p = 0.010). No quantitative caries or periodontal indices were reported.	Polypharmacy, with a mean of approximately 8 medications, was significantly associated with dry mouth (p = 0.036), feeling of no saliva (p = 0.009), and taste disturbances (p = 0.041). Greater oral dryness was also associated with mucosal burning (p = 0.026) and swallowing difficulty (p = 0.037). Dryness severity appeared to be related to the overall number of medications rather than to any specific drug class.
Brukienė et al., 2011	Salivary flow, salivary pH, and buffering capacity were assessed. Dental plaque was evaluated using the Plaque Index and Plaque Formation Rate Index. Compared with healthy younger controls, institutionalized older adults showed poorer salivary protective function and higher plaque accumulation.	Medication use was obtained from institutional records, with a mean of 5.3 medications per participant, and was analyzed as part of overall health burden. Medication-specific effect estimates were not reported in sufficient detail, likely because of the pilot design and small sample size.
Janssens et al., 2017	41.4% of residents were dentate, with a mean of approximately 6 remaining teeth across the full sample, whereas 58.6% were edentulous. Among dentate residents, the median numbers of decayed and filled teeth were 2 and 4, respectively. The overall treatment index was low relative to the extent of missing teeth, suggesting substantial cumulative tooth loss.	Higher medication count and a higher dry-mouth risk score were associated with fewer remaining natural teeth (p = 0.005 for xerogenic medication burden). In heavily medicated residents, the proportion of untreated caries was lower and the treatment index was higher (p < 0.01), which may reflect earlier tooth loss or extraction in those with greater medication burden. No direct association was reported between medication count and current periodontal status.

3

## Data Availability

The datasets used and/or analyzed during the current study are available from the corresponding author.
